# Multistate reptile‐ and amphibian‐associated salmonellosis outbreaks in humans, United States, 2009–2018

**DOI:** 10.1111/zph.12990

**Published:** 2022-08-10

**Authors:** Michelle A. Waltenburg, Ariana Perez, Zainab Salah, Beth E. Karp, Jean Whichard, Beth Tolar, Lauren Gollarza, Lia Koski, Anna Blackstock, Colin Basler, Megin Nichols

**Affiliations:** ^1^ Division of Foodborne, Waterborne and Environmental Diseases Centers for Disease Control and Prevention Atlanta Georgia USA; ^2^ General Dynamics Information Technology Atlanta Georgia USA; ^3^ Division of Viral Diseases Centers for Disease Control and Prevention Atlanta Georgia USA; ^4^ CAITTA, Inc. Herndon Virginia USA; ^5^ Maricopa County Department of Public Health Phoenix Arizona USA; ^6^ Division of Scientific Education and Professional Development Centers for Disease Control and Prevention Atlanta Georgia USA; ^7^ One Health Office Centers for Disease Control and Prevention Atlanta Georgia USA

**Keywords:** amphibians, antimicrobial resistance, microbial, reptiles, salmonella, zoonotic diseases

## Abstract

Non‐typhoidal *Salmonella* cause an estimated 1.4 million human illnesses, 26,000 hospitalizations and 400 deaths annually in the United States. Approximately 11% of these infections are attributed to animal contact. Reptiles and amphibians are known sources of salmonellosis; young children (aged <5 years) are disproportionately affected by reptile‐ and amphibian‐associated salmonellosis (RAAS) outbreaks. We describe multistate RAAS outbreaks to characterize illnesses and inform prevention efforts. RAAS outbreaks were defined as ≥2 culture‐confirmed human *Salmonella* infections with similar pulsed‐field gel electrophoresis patterns and epidemiologic, laboratory or traceback evidence linking them to a common reptile/amphibian exposure. Data sources included the Animal Contact Outbreak Surveillance System; CDC Outbreak Response and Prevention Branch's outbreak management database; PulseNet, the national molecular subtyping network for foodborne disease surveillance in the United States; and the National Antimicrobial Resistance Monitoring System. Twenty‐six RAAS outbreaks were reported during 2009–2018, resulting in 1465 illnesses and 306 hospitalizations. The outbreaks were associated with turtles (19), lizards (5), snakes (1) and frogs (1). Sixteen (61.5%) outbreaks were linked to small turtles (<4 inches), resulting in 914 illnesses. Forty‐nine percent of outbreak‐associated patients were aged <5 years. Of 362 patients/caregivers interviewed, 111 (30.7%) were aware that reptiles/amphibians can carry *Salmonella*. Among 267 patient isolates with antimicrobial susceptibility information, 20 (7.5%) were non‐susceptible to ≥1 antibiotic used to treat human salmonellosis. RAAS outbreaks result in considerable morbidity, particularly among young children. Illnesses linked to small turtles are preventable through education, targeted outreach to caregivers and paediatricians, and when appropriate, enforcement. Historically, individual states and jurisdictions have enforced existing or promulgated new authorities to address outbreaks. Preventing future RAAS outbreaks requires addressing challenges related to the illegal sale/distribution of small turtles; and for legal reptile sales, providing information on RAAS risk to consumers at point of sale to support informed pet ownership decisions.

**Impacts**

- Reptile and amphibian contact remains an important source of salmonellosis in the United States, with young children disproportionately affected by reptile‐ and amphibian‐associated salmonellosis (RAAS) outbreaks.
- Most multistate RAAS outbreaks and outbreak‐associated illnesses were linked to small turtles. Occurrence of these preventable outbreaks highlights challenges with enforcing regulations prohibiting their sale/distribution as pets.
- Non‐susceptibility to clinically important antibiotics in *Salmonella* causing RAAS outbreaks is a public health concern because it can make severe infections harder to treat.
- To prevent future RAAS outbreaks, information on RAAS risks should be readily available to consumers at point of sale to support informed pet ownership decisions.

## 1 INTRODUCTION

Non‐typhoidal *Salmonella* cause an estimated 1.4 million human illnesses, 26,000 hospitalizations and 400 deaths annually in the United States (Collier et al., 2021). *Salmonella* infection typically causes a self‐limiting gastroenteritis (e.g. diarrhoea, fever and abdominal pain); however, young children (<5 years of age), persons ≥65 years of age and immunocompromised individuals are at greater risk for serious complications, including septicaemia, meningitis and death (Wen et al., 2017).

Although commonly transmitted through food or water, an estimated 11% of all non‐typhoidal *Salmonella* infections are attributed to animal contact (Hale et al., 2012). Contact with reptiles and amphibians has resulted in multistate reptile‐ and amphibian‐associated salmonellosis (RAAS) outbreaks, which disproportionately affected young children (aged <5 years) (Harris et al., 2010; Kiebler et al., 2020; Mettee Zarecki, 2013; Vora et al., 2012; Whitten et al., 2015). Historically, *Salmonella* infections linked to pet turtles were so common that a federal ban on the sale of small turtles (<4 inches) was implemented in 1975 (Food and Drug Administration, 2019). The prevalence of *Salmonella* carriage can be >90% in reptiles and amphibians, and studies have documented *Salmonella* detection from pet reptiles and surfaces in reptile‐owning households (Back et al., 2016; Clancy et al., 2016; Lowther et al., 2011; Nakadai et al., 2005). Approximately 2.9% of U.S. households owned reptiles in 2016, representing a 17% increase in reptile ownership since 2011 (American Veterinary Medical Association, 2018).

Although most non‐typhoidal *Salmonella* infections do not warrant antibiotic treatment, administration of antibiotics is recommended to treat severe infections such as bacteraemia and meningitis and may benefit patients at increased risk for invasive infection (Pegues & Miller, 2015). Antimicrobial resistance has been identified among *Salmonella* isolated from pet reptiles and amphibians and from people linked to RAAS outbreaks, raising concern regarding the potential for these pets to serve as sources of resistant bacteria resulting in human infection (Centers for Disease Control and Prevention, 2014; Centers for Disease Control and Prevention, 2016a; Chen et al., 2010; Guerra et al., 2010).

We aimed to describe the epidemiology of multistate RAAS outbreaks in the United States from 2009 to 2018. We quantified the number of outbreaks and associated cases, and described the trends, patient demographics, types of human‐animal interactions most likely to lead to infection, and antimicrobial susceptibility of strains causing RAAS outbreaks.

## 2 MATERIALS AND METHODS

### 2.1 Outbreak detection and case identification

We defined multistate RAAS outbreaks as outbreaks with ≥2 culture‐confirmed human *Salmonella* infections with exposures reported from ≥2 states or territories with similar pulsed‐field gel electrophoresis (PFGE) patterns and with a combination of epidemiologic, laboratory and traceback evidence linking them to contact with a common reptile or amphibian species during 2009–2018 (Centers for Disease Control and Prevention, 2020). We defined reptile or amphibian contact as contact with the animal, its bodily fluids, food or contact with the environment where the animal resides. For each outbreak, we defined a case as an illness in a person infected with a laboratory‐confirmed *Salmonella* isolate matching the outbreak PFGE pattern and occurring while the outbreak investigation was ongoing (date of first patient illness onset to date outbreak investigation was declared over by CDC). Data sources included the Animal Contact Outbreak Surveillance System (ACOSS); CDC Outbreak Response and Prevention Branch's outbreak management database; PulseNet, the national laboratory network for molecular subtyping of *Salmonella* and other enteric pathogens; and the National Antimicrobial Resistance Monitoring System (NARMS). We reviewed data on reptile and amphibian exposures collected from patients (or their parents/guardians) during outbreak investigations.

### 2.2 Outbreak and patient characteristics

We examined outbreak characteristics and trends over time, including number of outbreaks per year, type of reptile or amphibian implicated, outbreak size and duration, specific *Salmonella* serotype(s), and isolates from specimens collected from animals or their environment (e.g. habitat, water, bedding). We classified reptile or amphibian type into five categories: turtles ≥4 inches, turtles <4 inches, lizards (e.g. bearded dragons, geckos), snakes and frogs. Isolates that had serotypes that were undetermined, pending or did not match the predominant outbreak strain were individually reviewed and determined to be consistent with the predominant outbreak serotypes based on PFGE pattern. Therefore, for the purposes of this analysis, these isolates were categorized as the predominant serotype if their PFGE pattern was indistinguishable from the outbreak PFGE pattern.

We examined patient characteristics including demographic information (e.g. age and sex), health outcomes (e.g. hospitalizations and deaths) and isolate source (e.g. stool, urine or blood). We defined infants as children <1 year of age. We considered the identification of an isolate from a blood sample as an indicator for a bloodstream infection. When patient characteristics were unknown, the data were considered missing for this analysis. Reported deaths were examined, and those not attributable to salmonellosis were excluded from this analysis. We compared outbreak and patient characteristics of small turtle outbreaks to all other RAAS outbreaks to examine differences by category of reptile or amphibian implicated. We analysed common pet ownership and handling practices reported among patients.

### 2.3 Antimicrobial susceptibility

Antimicrobial susceptibility testing (AST) was performed on a subset of patient, animal and environmental isolates using broth microdilution (Sensititre™, Thermo Fisher Scientific, Waltham, MA) according to manufacturer's instructions. We interpreted results using Clinical and Laboratory Standards Institute (CLSI) breakpoints, where available or NARMS consensus breakpoints for drugs without CLSI breakpoints (Centers for Disease Control and Prevention, 2016b; Clinical and Laboratory Standards Institute, 2020). Whole genome sequence (WGS) data was collected for select isolates by state public health laboratories or CDC using PulseNet standard methods and quality requirements (Centers for Disease Control and Prevention, 2016c). Antimicrobial resistance genes and mutations were identified from assembled sequences based on the ResFinder (updated May 23, 2019) and PointFinder (updated April 29, 2019) databases that detect acquired resistance genes and mutations, respectively (Bortolaia et al., 2020; Camacho et al., 2009; Center for Genomic Epidemiology, 2011; Hendriksen et al., 2019; McDermott et al., 2016; Zankari et al., 2017).

We defined isolates as non‐susceptible if they had a minimum inhibitory concentration (MIC) in the resistant or intermediate range by AST or a resistance mechanism (gene or mutation) identified by ResFinder and PointFinder screening of assembled WGS data; isolates with a *fosA7* gene and no other resistance mechanisms were considered susceptible. We defined clinically important antibiotics as those used to treat salmonellosis in people, including ampicillin, azithromycin, ceftriaxone, ciprofloxacin and trimethoprim‐sulfamethoxazole; isolates with non‐susceptibility (phenotypic or genotypic) to any of these agents were considered to have clinically important resistance (American Academy of Pediatrics, 2018; Shane et al., 2017).

### 2.4 Statistical methods

We determined the median and range for outbreak size and duration and calculated the mean number of outbreaks that occurred during two time periods: 2009–2013 and 2014–2018. We calculated frequencies for outbreak variables (category of reptile or amphibian implicated, *Salmonella* serotype) and patient variables (age, sex, hospitalizations, deaths, isolate source and pet ownership and handling practices). We described hospitalization and isolate source by patient age. We compared outbreak size, duration, median age and proportion of patients hospitalized by outbreak type (small turtle vs. all other RAAS outbreaks) using the Wilcoxon rank‐sum test and the Chi‐square test, respectively and resulting two‐sided *p*‐values. Analyses were conducted using SAS version 9.4 (SAS Institute, Cary, NC, USA).

## 3 RESULTS

### 3.1 Outbreak characteristics

**FIGURE 1:**
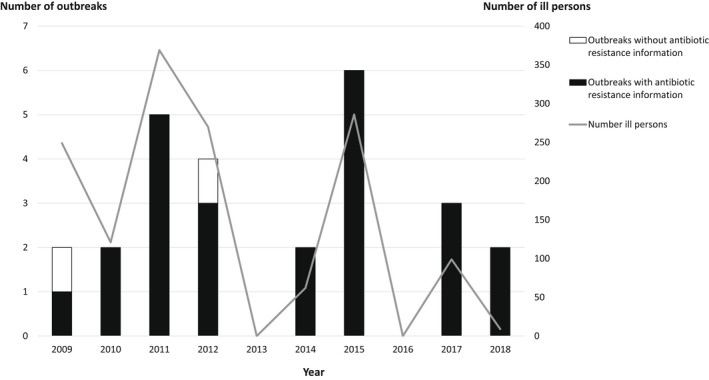
Number of multistate reptile‐ and amphibian‐associated salmonellosis outbreaks, number of ill persons and number of outbreaks with resistance data^†^ by year of first illness onset—United States, 2009–2018. ^†^Includes outbreaks with antimicrobial susceptibility testing data and/or resistance mechanism data from whole genome sequencing

A total of 26 multistate RAAS outbreaks were detected in the United States during 2009–2018, resulting in 1465 illnesses (Figure 1, Table 1). Sixteen (61.5%) RAAS outbreaks were epidemiologically linked to small turtles (<4 inches), five (19.2%) were linked to lizards (bearded dragons or geckos), three (11.5%) were linked to larger turtles (≥4 inches), one (3.8%) was linked to snakes, and one (3.8%) was linked to frogs (Table 1). Median outbreak size was 33 cases (range 3–238) and the median number of states involved was 13 (range 3–41). The median size of small turtle outbreaks was 51 cases (range 6–138) and the median size of all other RAAS outbreaks was 17 cases (range 3–238) (*p* = .246).

**TABLE 1:** Characteristics of multistate reptile‐ and amphibian‐associated salmonellosis outbreaks, by animal category—Animal contact outbreak surveillance system, United States, 2009–2018

Characteristic^a^	Turtle		Lizard		Snake	Frog	Total
	<4 inches	≥4 inches	Bearded dragon	Gecko			
Outbreaks, *n* (row%)	16 (61.5)	3 (11.5)	3 (11.5)	2 (7.8)	1 (3.8)	1 (3.8)	26 (100)
Ill persons, *n* (row%)	914 (62.4)	93 (6.3)	175 (11.9)	34 (2.3)	11 (0.8)	238 (16.2)	1465 (100)
Hospitalization^b^, *n* (col%)							
Yes	200 (31.1)	8 (20.5)	45 (36.6)	6 (20.0)	2 (18.2)	45 (31.0)	306 (30.8)
No	444 (68.9)	31 (79.5)	78 (63.4)	24 (80.0)	9 (81.8)	100 (69.0)	686 (69.2)
Missing	270	54	52	4	0	93	473
*Salmonella* serotype (no. of outbreaks)	Multiple serotypes (5)^c^ Sandiego (3) Agbeni (2) Pomona (2) Poona (2) I 4,[5],12:i:‐ (1) Typhimurium (1)	Muenchen (1) Poona (1) Paratyphi B var. L(+) tartrate+ and I 4,[5],12:b:‐ (1)	Apapa (1) Offa (1) Cotham and Kisarawe (1)	Fluntern (1) Muenchen (1)	Paratyphi B var. L(+) tartrate+ and Mbandaka (1)	Typhimurium (1)	—

^a^ Percentages might not total 100 due to rounding.

^b^ Variables are reported based on information available. Percentages were calculated without missing data. No deaths were reported among 990 patients; information on deaths was missing for 475 patients.

^c^ Five outbreaks consisted of multiple *Salmonella* serotypes: a 79‐person 2011 outbreak of *Salmonella Paratyphi* B var. L(+) tartrate+ and I 4,[5],12:b:‐; a 124‐person 2011 outbreak of *Salmonella* Sandiego and Newport; a 58‐person 2011 outbreak of *Salmonella* Poona and Sandiego; a 138‐person 2015 outbreak of *Salmonella* Poona, Pomona and Paratyphi B var. L(+) tartrate+; and a 6‐person 2015 outbreak of *Salmonella* Litchfield, Braenderup, IV 44:z4,z32:‐ and IIIb 61:i:z53.

The annual number of RAAS outbreaks ranged from 0 to 6 (Figure 1). During 2009–2013, a total of 13 outbreaks were reported (mean 2.6/year), resulting in 1009 illnesses; median outbreak size was 58 cases (range 7–238). During 2014–2018, 13 outbreaks were also reported (mean 2.6/year), resulting in 456 illnesses; median outbreak size was 21 cases (range 3–138). The highest number of RAAS outbreaks occurred in 2015, with 6 outbreaks. The five largest outbreaks occurred in 2009 (1), 2011 (2), 2012 (1) and 2015 (1) and resulted in a total of 786 illnesses; the two largest outbreaks were associated with frogs and lizards (bearded dragons). Outbreak duration ranged from <1 to 29 months, with a median duration of 12 months. The median duration for small turtle outbreaks was 511 days (range 111–821) compared to 131 days (range 11–861) for all other RAAS outbreaks (*p* = 0.054).

Twenty *Salmonella* serotypes were identified across the 26 outbreaks. Eight of 26 (30.8%) RAAS outbreaks consisted of multiple *Salmonella* serotypes (Table 1). Among single serotype RAAS outbreaks, Poona and Sandiego were each reported in three outbreaks, all of which were linked to turtles. The most common serotypes across all outbreak‐associated isolates were Typhimurium (282 cases), Poona (268 cases), Sandiego (190 cases), Pomona (179 cases), Cotham (160 cases), Agbeni (155 cases) and Paratyphi B var. L(+) tartrate+ (80 cases) (Table 2). The *Salmonella* serotypes with the highest proportion of blood isolates among all isolates of each respective serotype were Agbeni (20.6%, 32/155), Sandiego (13.2%, 25/189) and Pomona (11.8%, 21/178). Nine (34.6%) outbreaks included animal or environmental isolates from samples collected during outbreak investigations.

**TABLE 2:** Serotypes of *Salmonella* isolates from patients in multistate reptile‐ and amphibian‐associated salmonellosis outbreaks, by isolate source, United States, 2009–2018

Serotype^a^	No. isolates	No. isolates with specified source site	No. (%) stool	No. (%) urine	No. (%) blood	No. (%) other source^b^
Typhimurium	282	272	258 (94.9)	9 (3.3)	4 (1.5)	1 (0.4)
Poona	268	266	219 (82.3)	23 (8.6)	21 (7.9)	3 (1.1)
Sandiego	190	189	135 (71.4)	24 (12.7)	25 (13.2)	5 (2.6)
Pomona	179	178	135 (75.8)	18 (10.1)	21 (11.8)	4 (2.2)
Cotham	160	157	121 (77.1)	19 (12.1)	14 (8.9)	3 (1.9)
Agbeni	155	155	101 (65.2)	20 (12.9)	32 (20.6)	2 (1.3)
Paratyphi B var. L(+) tartrate+	80	79	73 (92.4)	3 (3.8)	3 (3.8)	0 (0)
I 4,[5],12:b:‐	59	59	48 (81.4)	6 (10.2)	5 (8.5)	0 (0)
Muenchen	33	32	25 (78.1)	5 (15.6)	1 (3.1)	1 (3.1)
I 4,[5],12:i:‐	19	18	18 (100)	0 (0)	0 (0)	0 (0)
Fluntern	12	10	10 (100)	0 (0)	0 (0)	0 (0)
Other serotype^c^	28	28	23 (82.1)	3 (10.7)	1 (3.6)	1 (3.6)
Total	1,465	1,443	1166 (80.8)	130 (9.0)	127 (8.8)	20 (1.4)

^a^ Percentages might not total 100 due to rounding.

^b^ Other sources included skin, gall bladder, abscess, respiratory, cerebrospinal fluid or other tissue or body fluid.

^c^ Other *Salmonella* serotypes isolated from patients included: Apapa (6 cases; bearded dragon), Kisarawe (6 cases; bearded dragon), Mbandaka (6 cases; snake), Litchfield (3 cases; small turtle), Offa (3 cases; bearded dragon), Braenderup (1 case; small turtle), IIIb 61:i:z53 (1 case; small turtle), IV 44:z4,z32:‐ (1 case; small turtle) and Newport (1 case; small turtle).

### 3.2 Patient characteristics

**FIGURE 2:**
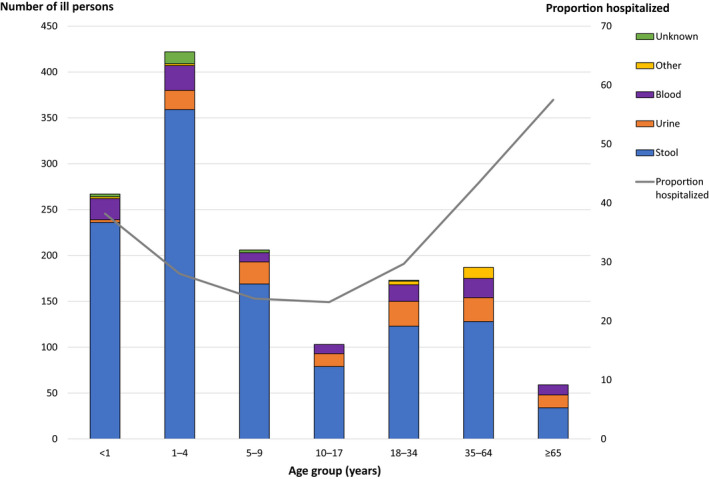
Number of ill persons, proportion hospitalized and specimen sources in multistate reptile‐ and amphibian‐associated salmonellosis outbreaks, by age group—United States, 2009–2018.^†^^†^Information on age was available for 1417 ill persons

Among patients with available demographic information, 54.8% (771/1406) were female. More than two‐thirds of patients (70.4%, 998/1417) were aged <18 years: 18.8% (267/1417) of patients were infants, 29.8% (422/1417) were children 1–4 years, and 14.5% (206/1417) were children 5–9 years (Figure 2). The overall median patient age was 5 years (range <1–100 years). Among patients with information on health outcomes, 30.8% (306/992) were hospitalized and no deaths were reported. Among 290 hospitalized patients with known age, the groups with the highest number of patients hospitalized were infants (73), children 1–4 years (73) and patients 35–64 years (46). Hospitalization rates were highest among patients ≥65 years (57.5%, 23/40), patients 35–64 years (43.4%, 46/106), and infants (38.2%, 73/191) (Figure 2).

Among 1443 patients with available isolate source information, 1166 (80.8%) had *Salmonella* isolated from a stool specimen, 130 (9.0%) from urine, 127 (8.8%) from blood and 20 (1.4%) from other sites (Table 2). Patients ≥65 years had the highest proportion of *Salmonella* isolated from their blood (18.6%, 11/59) (Figure 2). Children 1–4 years had the greatest number of blood isolates (27/120; 22.5%).

More than 60% of all RAAS outbreak‐associated illnesses were linked to small turtles (Table 1). Overall, 69.2% (608/878) of patients in small turtle outbreaks were <18 years of age. The median patient age (5 years) and age range (<1–100 years) did not differ substantially between small turtle outbreaks and all other RAAS outbreaks (*p* = .083). Among all patients in RAAS outbreaks with known hospitalization status (992/1465; 67.7%), the proportion of patients hospitalized across all small turtle outbreaks was 31.1% compared to 30.4% across all other RAAS outbreaks (*p* = .846).

Reptile and amphibian exposure questionnaires were administered to 429 (29.3%) patients from 23 (88.5%) outbreaks; of these, 311 (72.5%) reported contact with a reptile or amphibian in the 7 days prior to illness onset. Among 309 people who specified contact location, 77.3% (239/309) reported that contact occurred in the home. Of the 208 respondents who specified the type of contact they had with the reptile or amphibian, 12.5% (26/208) reported direct contact (touching, holding, kissing), 38.5% (80/208) reported indirect contact (feeding, watering and habitat contact), and 49.0% (102/208) reported both direct and indirect contact. Nearly 30.0% (55/184) of respondents reported allowing the reptile or amphibian to roam freely outside of its cage or enclosure. When asked if they were aware of a connection between reptile or amphibian contact and *Salmonella*, only 30.7% (111/362) of respondents reported that they were aware. A higher proportion of bearded dragon (73.3%) and snake (80.0%) owners were aware of a connection between their pet and *Salmonella* compared to turtle owners (17.1%).

### 3.3 Antimicrobial susceptibility

Antibiotic susceptibility information was available for 267 (18.2%) patient isolates from 24 (92.3%) outbreaks; 84 isolates were screened for antibiotic non‐susceptibility by phenotypic AST, 138 by WGS and 45 by both methods (Figure 1, Table 3). Twenty‐one (7.9%) patient isolates from nine outbreaks were non‐susceptible to at least one antibiotic tested. Among these 21 patient isolates, 20 (95.2%) were non‐susceptible to one or more clinically important antibiotics; of these, eight isolates were non‐susceptible to two clinically important antibiotics and one was non‐susceptible to three clinically important antibiotics. Additionally, antibiotic non‐susceptibility information was available for 10.9% (39/359) of animal and environmental (e.g. habitat, water and bedding) isolates from nine outbreaks. One (2.6%) isolate, an environmental isolate from the snake outbreak, was non‐susceptible to a clinically important antibiotic. Various resistance mechanisms that confer non‐susceptibility to fluoroquinolones, β‐lactam antibiotics and trimethoprim‐sulfamethoxazole were identified in isolates with WGS data (Table 4).

**TABLE 3:** Antimicrobial nonsusceptibility among patient isolates from multistate reptile‐ and amphibian‐associated salmonellosis outbreaks, by reptile/amphibian category—United States, 2009–2018

Category of reptile or amphibian implicated	No. (%) of outbreaks with nonsusceptibility detected^a^	No. (%) of non‐susceptible isolates
Turtle		
<4 inches	4/15 (26.7)	8/184 (4.3)
≥4 inches	0/2 (0)	0/7 (0)
Lizard		
Bearded dragon	3/3 (100)	8/23 (34.8)
Gecko	0/2 (0)	0/19 (0)
Snake	1/1 (100)	4/10 (40.0)
Frog	1/1 (100)	1/24 (4.2)
Total	9/24 (37.5)	21/267 (7.9)

^a^ Includes outbreaks and patient isolates with antimicrobial susceptibility testing (AST) data or resistance mechanism data from whole genome sequencing (WGS). No isolates were screened for susceptibility in two outbreaks: an 11‐person 2009 outbreak of *Salmonella* Muenchen that was associated with turtles (≥4 inches) and a 19‐person 2012 outbreak of *Salmonella* I 4,[5],12:i:‐ associated with small turtles (<4 inches). AST was performed using broth microdilution. We used Clinical and Laboratory Standards Institute breakpoints (where available) or consensus breakpoints from the National Antimicrobial Resistance Monitoring System. Whole genome sequence data was collected for select isolates by state public health laboratories or CDC using PulseNet standard methods and quality requirements. Antimicrobial resistance genes and mutations were identified from assembled sequences based on the ResFinder (updated May 23, 2019) and PointFinder (updated April 29, 2019) databases. We defined isolates as non‐susceptible if they had a minimum inhibitory concentration in the resistant or intermediate range by AST or a resistance mechanism by WGS; isolates with a *fosA7* gene and no other resistance mechanism were considered susceptible.

**TABLE 4:** Antimicrobial susceptibility profiles and resistance mechanisms detected among patient isolates from multistate reptile‐ and amphibian‐associated salmonellosis outbreaks —United States, 2009–2018

Outbreak year	Type of reptile or amphibian implicated	*Salmonella* serotype	No. of ill persons	% isolates non‐susceptible^a^ (no. non‐susceptible /no. screened)	Antimicrobial non‐susceptibility profile^a^	Resistance mechanism(s) detected
2009	Frog	Typhimurium	238	4.2 (1/24)	23 susceptible 1 ASSuTAuCxFoxKan	5 none
2009	Turtle (≥4 inches)	Muenchen	11	NA	NA	NA
2010	Turtle (≥4 inches)	Paratyphi B var. L(+) tartrate+	21	0 (0/1)	1 susceptible	NA
		I 4,[5],12:b:‐	21	0 (0/4)	4 susceptible	NA
2010	Turtle (<4 inches)	Paratyphi B var. L(+) tartrate+	41	0 (0/5)	5 susceptible	NA
		I 4,[5],12:b:‐	38	NA	NA	NA
2011	Turtle (<4 inches)	Sandiego	123	0 (0/8)	8 susceptible	1 none
		Newport	1	NA	NA	NA
2011	Turtle (<4 inches)	Pomona	23	0 (0/3)	3 susceptible	1 none
2011	Turtle (<4 inches)	Pomona	120	0 (0/8)	8 susceptible	1 none
2011	Turtle (<4 inches)	Typhimurium	44	0 (0/9)	9 susceptible	NA
2011	Turtle (<4 inches)	Poona	36	0 (0/5)	5 susceptible	2 none
		Sandiego	22	NA	NA	NA
2012	Lizard (bearded dragon)	Cotham	160	7.1 (1/14)	13 susceptible 1 ACSSuTAuCxFoxCipGen	4 none 1 *aac(3)‐IId aph(3″)‐Ib aph(6)‐Id bla*_CMY‐2_ *floR qnrS1 sul2 tet(A)*
		Kisarawe	6	0 (0/1)	1 susceptible	1 none
2012	Turtle (<4 inches)	Sandiego	7	0 (0/3)	3 susceptible	NA
2012	Turtle (<4 inches)	Poona	78	0 (0/3)	3 susceptible	NA
2012	Turtle (<4 inches)	I 4,[5],12:i:‐	19	NA	NA	NA
2014	Turtle (≥4 inches)	Poona	40	0 (0/2)	2 susceptible	NA
2014	Lizard (crested gecko)	Muenchen	22	0 (0/8)	8 susceptible	7 none
2015	Turtle (<4 inches)	Pomona	36	3.3 (1/30)	29 susceptible 1 A	29 none 1 *bla*_TEM‐116_
		Poona	89	11.5 (3/26)	23 susceptible 1 A 1 AAu 1 T	19 none 1 *bla*_TEM‐1C_ 1 *bla*_TEM‐1B_ 1 *tet(B)*
		Paratyphi B var. L(+) tartrate+	13	NA	NA	NA
2015	Turtle (<4 inches)^b^	Sandiego	17	0 (0/3)	3 susceptible	3 none
2015	Turtle (<4 inches)	Poona	25	0 (0/6)	6 susceptible	3 none
2015	Turtle (<4 inches)	Sandiego	21	50.0 (1/2)	1 susceptible 1 ASTGen^c^	1 none
2015	Turtle (<4 inches)	Agbeni	79	8.7 (2/23)	21 susceptible 2 AAuCxFox	21 none 2 *bla*_CMY‐2_
2015	Turtle (<4 inches)	IIIb 61:i:z53	1	0 (0/1)	1 susceptible	1 none
		Braenderup	1	NA	NA	NA
		Litchfield	3	NA	NA	NA
		IV 44:z4,z32:‐	1	NA	NA	NA
2017	Turtle (<4 inches)	Agbeni	76	2.0 (1/49)	48 susceptible 1 CipNal	48 none 1 *gyrA*(83)
2017	Snake^d^	Paratyphi B var. L(+) tartrate+	5	100.0 (4/4)	3 CipNal 1 CCipNal^e^	4 *gyrA*(87)
		Mbandaka	6	0 (0/6)	6 susceptible	6 none
2017	Lizard (leopard gecko)^f^	Fluntern	12	0 (0/11)	11 susceptible	10 none
2018	Lizard (bearded dragon)	Offa	3	66.7 (2/3)	1 susceptible 2 Cip	1 none 2 *qnrB19*
2018	Lizard (bearded dragon)	Apapa	6	100.0 (5/5)	5 SSuCipCotTmpFos	4 *aadA1 aadA2 aph(3″)‐Ib aph(6)‐Id dfrA12 fosA7 qnrB19 sul1 sul3* 1 *aadA2 ant(3″)‐Ia aph(3″)‐Ib aph(6)‐Id dfrA12 fosA7 qnrB19 sul1 sul3*

Abbreviations: A, ampicillin; Au, amoxicillin‐clavulanic acid; C, chloramphenicol; Cip, ciprofloxacin; Cot, trimethoprim‐sulfamethoxazole; Cx, ceftriaxone; Fos, fosfomycin; Fox, cefoxitin; Gen, gentamicin; Kan, kanamycin; NA, no isolates screened or sequenced; Nal, nalidixic acid; S, streptomycin; Su, sulfisoxazole; T, tetracycline; Tmp, trimethoprim.

^a^ Includes patient isolates with antimicrobial susceptibility testing (AST) data or resistance mechanism data from whole genome sequencing (WGS). AST was performed using broth microdilution. We used Clinical and Laboratory Standards Institute breakpoints (where available) or consensus breakpoints from the National Antimicrobial Resistance Monitoring System. Whole genome sequence data was collected for select isolates by state public health laboratories or CDC using PulseNet standard methods and quality requirements. Antimicrobial resistance genes and mutations were identified from assembled sequences based on the ResFinder (updated May 23, 2019) and PointFinder (updated April, 29 2019) databases. We defined isolates as non‐susceptible if they had a minimum inhibitory concentration in the resistant or intermediate range by AST or a resistance mechanism by WGS.

^b^ Three isolates with a *fosA7* gene and no other resistance mechanism were considered susceptible.

^c^ Plasmid loss likely occurred in this isolate, which had no resistance mechanisms detected by WGS and was susceptible on retesting.

^d^ One environmental isolate also had *gyrA*(87).

^e^ This isolate was chloramphenicol intermediate but had no detected phenicol resistance mechanism.

^f^ Ten isolates with a *fosA7* gene and no other resistance mechanism were considered susceptible.

Patient isolates were most often non‐susceptible to the fluoroquinolone ciprofloxacin. Thirteen (4.9%) patient isolates and one (2.6%) environmental isolate had a quinolone‐resistance mechanism (Table 4). Eight of the 13 patient isolates were from three bearded dragon outbreaks and carried a plasmid‐mediated quinolone‐resistance (PMQR) gene (seven *qnrB19*, one *qnrS1*). The remaining five patient isolates had a quinolone resistance‐determining region (QRDR) mutation; of these, one isolate from a small turtle outbreak had a *gyrA*(83) mutation and four isolates from the snake outbreak had a *gyrA*(87) mutation. The non‐susceptible environmental isolate from the snake outbreak had the same *gyrA*(87) mutation as the human isolates from the outbreak.

Phenotypic or genotypic non‐susceptibility to β‐lactam antibiotics was noted among eight patient isolates (Table 4). Four patient isolates from three outbreaks (turtle <4 inches, bearded dragon and frog) were non‐susceptible to ceftriaxone, ampicillin, cefoxitin and amoxicillin‐clavulanic acid; of these, three isolates were sequenced and found to have the AmpC β‐lactamase gene *bla*_CMY‐2_. Four additional patient isolates from two small turtle outbreaks were non‐susceptible to ampicillin, including one isolate, which was also non‐susceptible to amoxicillin‐clavulanic acid. Three of these isolates had *bla*_TEM_ genes.

Five patient isolates from one bearded dragon outbreak were non‐susceptible to trimethoprim‐sulfamethoxazole; all five isolates were sequenced and found to have *dfrA12, sul1* and *sul3* genes. Azithromycin non‐susceptibility was not found among patient isolates. Non‐susceptibility to other antimicrobials (e.g. aminoglycosides, phenicols and tetracyclines) was noted in some isolates (Table 4).

## 4 DISCUSSION

Reptile‐ and amphibian‐associated salmonellosis outbreaks resulted in considerable morbidity, particularly among young children. These outbreaks are a reminder that contact with reptiles and amphibians remains an important source of salmonellosis in the United States. Consistent with historical outbreaks of *Salmonella* linked to animal contact, RAAS outbreaks in this analysis were longer in duration compared to foodborne *Salmonella* outbreaks (Marshall et al., 2020; Marus et al., 2019). Reptiles and amphibians can be colonized with multiple strains of *Salmonella*, appear healthy while colonized and shed *Salmonella* intermittently; people might be exposed long after obtaining their pet, which can result in prolonged outbreak durations (Goupil et al., 2012; Hoelzer et al., 2011; Whitten et al., 2015). Compared to all patients with non‐typhoidal *Salmonella* infections (Jones et al., 2008), patients in RAAS outbreaks also had higher proportions of bloodstream infections (8.8% vs. 5%) and hospitalizations (30.8% vs. 22%). These findings indicate that infections associated with RAAS outbreaks continue to result in more adverse outcomes compared to other non‐typhoidal *Salmonella* infections and outbreaks (Marus et al., 2019). Previous studies have reported blood isolation to be more common in serotype Sandiego than Typhimurium; however, this has not been previously reported for serotype Agbeni, as we found in this analysis (Angelo et al., 2016; Jones et al., 2008). Serotype Agbeni is an uncommon serotype in humans that appears to be emerging in the United States (Centers for Disease Control and Prevention, 2018).

Although a federal ban on the sale of small turtles was implemented in 1975 (Food and Drug Administration, 2019), small turtles were the most common reptile/amphibian vehicle implicated in multistate RAAS outbreaks that occurred during 2009–2018. Consistent with other RAAS outbreaks, small turtle outbreaks were prolonged and disproportionately affected children. Over 60% of outbreaks and more than 900 illnesses linked to small turtles could have been avoided if the sale and distribution of small turtles as pets had been prevented. Small turtles are still available for legal purchase online for scientific, educational or exhibition purposes and might be illegally sold primarily through transient street vendors, at flea markets and at fairs (Basler et al., 2015; Food and Drug Administration, 2019). Unless sales are prevented, including through enforcement of existing regulations and consumer education, illnesses and outbreaks may continue to occur. Tracing the source of purchase locations can be difficult in small turtle outbreaks because of the transient nature of some small turtle vendors, which might pose challenges to turtle ban enforcement (Walters et al., 2016). Public health, veterinary and regulatory officials should continue to investigate these outbreaks to identify illegal sales and prevent additional illnesses associated with small turtles.

Children continue to be disproportionately impacted by RAAS outbreaks. While preventive measures can be implemented to safely interact with pet reptiles and amphibians, such as frequent and thorough hand washing (Centers for Disease Control and Prevention, 2019), children tend to have increased hand‐to‐face behaviour and it might be difficult to ensure that they immediately and properly wash their hands (McMillian et al., 2007). A high proportion of outbreak‐associated illnesses occurred in infants who might be less likely to handle reptiles or amphibians directly, suggesting that the presence of these pets in the household poses a risk of *Salmonella* transmission, regardless of direct pet contact and interaction (Lowther et al., 2011; Walters et al., 2016). Owners should take steps to mitigate indirect transmission, such as not allowing pets to roam freely in the house and keeping pets and items from the pet's habitat out of the kitchen and other areas where food is consumed, stored or prepared (Centers for Disease Control and Prevention, 2019; Lowther et al., 2011). Our findings reinforce public health recommendations that reptiles and amphibians should not be kept in homes or schools/day care settings with young children (Centers for Disease Control and Prevention, 2019).

The emergence of antibiotic‐resistant *Salmonella* in pet reptiles and amphibians is a growing public health concern and has implications for both human and veterinary medicine. Although antimicrobials are indicated to treat certain diseases in reptiles and amphibians, it is not recommended to treat healthy reptiles and amphibians with antimicrobials with the intention of eliminating *Salmonella* species from the intestinal tract (Association of Reptile and Amphibian Veterinarians, 2016; Bradley & Angulo, 2009; Clancy et al., 2016; Smith, 2007). Historically, antimicrobials such as oxytetracycline, gentamicin and enrofloxacin were used in attempts to reduce or eliminate *Salmonella* from reptiles through the treatment of water and/or egg washes. As a result of development of antimicrobial‐resistant strains of *Salmonella* from water/egg wash treatments, current recommendations advise against the use of antimicrobials in reptiles to eliminate *Salmonella* (Association of Reptile and Amphibian Veterinarians, 2016; Mitchell & Shane, 2001). Current practices to reduce or eliminate *Salmonella* in reptiles include using non‐antibiotic compounds to treat water and/or eggs (Mitchell et al., 2007). Further evaluation to better understand drivers of antimicrobial resistance in pet reptiles and amphibians is needed, including antimicrobial use practices in the animal industry and spread of resistant bacteria on turtle farms.

We described antimicrobial non‐susceptibility among outbreak‐associated isolates and found that 7.5% of patient isolates were non‐susceptible to ≥1 antibiotic used to treat salmonellosis, including ciprofloxacin and ceftriaxone. Non‐susceptibility to ciprofloxacin among patient isolates is noteworthy, as studies suggest that even small increases in quinolone MICs can result in poorer clinical outcomes and facilitate the emergence of fluoroquinolone resistance (Crump et al., 2003; Humphries et al., 2012; Rodríguez‐Martínez et al., 2016). We identified PMQR genes among patient isolates, which is consistent with a recently published US study that identified PMQR genes in *Salmonella* isolates from patients with exposure to reptiles and amphibians (Karp et al., 2018). This finding is notable as PMQR genes may be transferred horizontally to other bacteria, thereby spreading resistance, and they can also facilitate the development of higher‐level quinolone resistance (Robicsek et al., 2006; Rodríguez‐Martínez et al., 2016). Some patient isolates were non‐susceptible to ceftriaxone, which is frequently recommended for treatment of invasive *Salmonella* infections, particularly in children (American Academy of Pediatrics, 2018).

These findings highlight the need for increased efforts to educate pet owners about the risk of infection from pet reptiles and amphibians. Because most contact occurred at home, pet owners need to be aware of steps to safely interact with their pets, including proper habitat cleaning. The lack of *Salmonella* awareness among pet turtle owners highlights a gap in education, especially when reptiles are sold and distributed illegally, and no information is provided to potential owners regarding *Salmonella* risk and prevention. All owners, when purchasing or obtaining a pet reptile should receive information regarding animal husbandry, *Salmonella* prevention and the importance of routine veterinary care. Veterinarians are uniquely suited to provide information on prevention of disease and animal husbandry to pet owners. Furthermore, information on the risks associated with pet reptiles and amphibians should be readily available to consumers at the point of sale to support informed decision‐making on appropriate pet ownership. Enhanced collaboration between industry (e.g. breeders and retailers), paediatricians, veterinarians, animal health and safety agencies, and public health agencies is needed to facilitate outbreak prevention steps. Collaboration would also ensure consistent prevention messaging and dissemination of information, which can be tailored to those most at risk of developing severe infections from reptiles and amphibians, such as young children and those aged ≥65 years.

This report is subject to several limitations. First, multistate RAAS outbreaks may not have been captured if they were not detected by PulseNet or if reptile or amphibian exposure information was not obtained by public health officials investigating cases of salmonellosis. Additionally, while multistate outbreaks represent a small portion of all illnesses linked to animal contact, they provide unique data about outbreak sources and settings. Second, missing data for patient health outcomes (e.g. hospitalizations, deaths) were common; this information was missing for approximately 30% of patients. Third, antimicrobial susceptibility information was not available for approximately 80% of patient isolates and 90% of animal and environmental isolates; for many RAAS outbreaks, only a subset of isolates underwent susceptibility testing. For some outbreaks, this may have affected the estimates of non‐susceptibility reported and limited our ability to detect the diversity of non‐susceptibility patterns present. With the transition from PFGE to WGS as the primary method of surveillance and outbreak detection of *Salmonella* in 2019 (Besser et al., 2019; Gerner‐Smidt et al., 2019), data on antibiotic resistance is becoming more complete as more isolates undergo sequencing. Additionally, information regarding treatment failures is not routinely collected during outbreak investigations, limiting our ability to assess the extent to which resistance impacted the ability to treat severe infections. Finally, reptile and amphibian exposure questionnaires were not administered to every patient in each RAAS outbreak; patients who did not complete questionnaires might have had different types of reptile or amphibian contact. Routine sampling of reptiles at breeders, distributors and retailers would yield data regarding the types of *Salmonella* carried by reptiles in the United States and might allow for more timely hypothesis generation of potential sources of *Salmonella* infection during outbreak investigations.

Contact with reptiles and amphibians remains an important source of salmonellosis in the United States, with young children disproportionately affected by RAAS outbreaks. To prevent future RAAS outbreaks, information on RAAS risks should be readily available to consumers at the point of sale to support informed decision‐making on appropriate pet ownership. Despite a federal ban on their sale and distribution, small turtles continue to result in a high proportion of otherwise preventable outbreaks and illness. Addressing challenges related to the illegal sale and distribution of small turtles might result in decreased reptile‐associated morbidity, especially among young children.

## CONFLICT OF INTEREST

None to declare.

## ETHICAL APPROVAL

This activity was reviewed by CDC and was conducted consistent with applicable federal law and CDC policy: 45 C.F.R. part 46, 21 C.F.R. part 56; 42 U.S.C. Sect. 241(d); 5 U.S.C. Sect. 552a; 44 U.S.C. Sect. 3501 et seq.

## DISCLAIMER

The findings and conclusions in this report are those of the authors and do not necessarily represent the official position of the Centers for Disease Control and Prevention.

## Data Availability

The data that support the findings of this study are available from the corresponding author upon reasonable request.
